# Progress in the development of early diagnosis and a drug with unique pharmacology to improve cancer therapy

**DOI:** 10.1098/rsta.2008.0106

**Published:** 2008-07-21

**Authors:** A. Lehotzky, N. Tőkési, I. Gonzalez-Alvarez, V. Merino, M. Bermejo, F. Orosz, P. Lau, G.G. Kovacs, J. Ovádi

**Affiliations:** 1 Institute of Enzymology, Biological Research Centre, Hungarian Academy of SciencesKarolina út 29, 1113 Budapest, Hungary; 2 Department of Pharmaceutics and Pharmaceutical Technology, University of Valencia46010 Valencia, Spain; 3 Section of Developmental Genetics, National Institute of Neurological Disorders and Stroke, National Institutes of HealthBethesda, MD 20892, USA; 4 Institute of Neurology, Medical University of Vienna1097 Vienna, Austria

**Keywords:** cancer, anti-mitotic drug, KAR-2, bioavailability, anti-mitotic protein, TPPP/p25

## Abstract

Cancer continues to be one of the major health and socio-economic problems worldwide, despite considerable efforts to improve its early diagnosis and treatment. The identification of new constituents as biomarkers for early diagnosis of neoplastic cells and the discovery of new type of drugs with their mechanistic actions are crucial to improve cancer therapy. New drugs have entered the market, thanks to industrial and legislative efforts ensuring continuity of pharmaceutical development. New targets have been identified, but cancer therapy and the anti-cancer drug market still partly depend on anti-mitotic agents. The objective of this paper is to show the effects of KAR-2, a potent anti-mitotic compound, and TPPP/p25, a new unstructured protein, on the structural and functional characteristics of the microtubule system. Understanding the actions of these two potential effectors on the microtubule system could be the clue for early diagnosis and improvement of cancer therapy.

## 1. Cancer and microtubule system

Differentiation is a normal process of the maturation of cells by which they become progressively more specialized. This process of specialization for the cell comes at the expense of its breadth of potential. In cancer, the mature tumour cells resemble normal cells and tend to grow and spread at a slower rate than undifferentiated or poorly differentiated tumour cells, which lack the structure and function of normal cells and grow uncontrollably. The most suggestive behaviour of cancerous cells is continuous cell division, which usually originates from one cell. The reasons behind this behaviour could be different and could originate from different parts of the cell cycle regulation. Failure or mis-control of the cell cycle can turn a cell tumorous and finally lead to cancer. In that case, the division of the cancerous cells proceeds in an uncontrolled manner and the degree of anaerobic glycolysis is very high to support the extra demand of energy for the continuously dividing cells. The event of mitosis is tightly coupled with the dynamic behaviour of the microtubules required for the reorganization of the microtubule system during the cell cycle; in fact, the microtubules are organized in radically different ways during the interphase and during mitosis. This reorganization results from duplication of the microtubule organizing centres, and from a profound shift in the pattern of microtubule dynamics (Altmann & Gertsch 2007; Bhat & Setaluri 2007; Sudakin & Yen 2007 and references therein).

Microtubules are polymers of α- and β-tubulin dimers. They are ubiquitous cellular structures involved in several essential cellular functions such as the extension and guidance of neurons at the growth cone, maintaining the shape of the cells, RNA transport and motility, and the formation of the mitotic spindle required for chromosomal segregation (MacRae 1992). Tubulin has more isoforms and could bear a huge range of post-translational modifications depending on the cellular environment (Luduena 1998; Dutcher 2001). The function of microtubules in cells is further supported and modified by microtubule-associated proteins (MAPs), which are usually expressed in a tissue-specific manner, colouring further the function and behaviour of the microtubule system. Microtubules move vesicles, granules, organelles such as mitochondria, and chromosomes via special attachment proteins. The multifarious behaviour of the microtubule system throughout the life of eukaryotic cells is ensured by its decoration by macromolecules, post-translational modifications, nucleotides and other small molecules. The discovery of new tubulin-binding constituents is crucial for understanding the cellular machinery controlled by the microtubule system.

## 2. TPPP/p25 and KAR-2 are new tubulin-binding constituents

We recently identified TPPP/p25, a brain-specific intrinsically unfolded protein, which primarily targets tubulin/microtubules in normal and cancer cells. TPPP/p25 promotes tubulin polymerization (which is why we denoted it as tubulin polymerization-promoting protein of 25 kDa) into intact or aberrant microtubules and aggregates (Hlavanda *et al*. 2002; Tirián *et al*. 2003). This protein displays a very high bundling activity causing extensive cross-linking of the polymerized microtubules. On the basis of these and some additional data, we suggested TPPP/p25 to be ranked into the family of MAPs (Vincze *et al*. 2006).

We also demonstrated that TPPP/p25 is enriched and colocalized with tubulin and α-synuclein in the Lewy bodies of Parkinson's disease and other inclusions, including those in oligodendroglial cells, a characteristic for synucleinopathies (Kovács *et al*. 2004; Orosz *et al*. 2004). By contrast, TPPP/p25 is not associated with abnormally phosphorylated tau, a feature of tauopathies such as Alzheimer's disease. All these studies, in which we used highly specific anti-TPPP/p25 polyclonal antisera (Kovács *et al*. 2004; Orosz *et al*. 2004), revealed that TPPP/p25 can be used as a marker to identify specific group of diseases. The relation of TPPP/p25 with the microtubule system and cell division process (Tirián *et al*. 2003) motivated us to investigate its role in other diseases such as cancer.

The microtubule system represents one of the best anti-cancer drug targets identified so far, and bis-indoles, a large group of anti-cancer agents, have been recognized as potent anti-mitotic, anti-proliferative agents. Their use in clinical chemotherapy, however, is limited owing to their undesirable toxic side effects such as neurological disorder. While screening a number of bis-indole molecules synthesized by the chemists of Richter Chemical Work Ltd, we identified a group of vinblastine derivatives (KARs) as potential anti-mitotic agents, and characterized the desired and undesired effects of KAR-2, as a lead molecule at different levels of organization (Orosz *et al*. 1999).

The cause of the toxicity of bis-indoles (vinblastine, vincristine, navelbine, etc.) is complex; nevertheless, there are two issues that are specifically considered in this work. First, the non-specific interaction of bis-indoles with mitotic (spindle) microtubules results in the disassembly of the interface microtubule network also in non-cancerous cells. Second, these drugs interact with intracellular proteins in addition to tubulin. For example, calmodulin, a multifarious Ca^2+^ receptor protein associated to enzymes with various functions and an integrant constituent of the cytoskeleton, has been identified as a potential target of the bis-indole derivatives (Orosz *et al*. 1997*b*). Recently, vinflunine, a novel semi-synthetic bis-indole with anti-mitotic activity, was found to suppress the interaction of calmodulin with the MAP STOP; in addition, different binding modes to calmodulin were reported for vinflunine and vinblastine (Makarov *et al*. 2007). Interestingly, KAR-2, as other bis-indoles, binds to calmodulin; however, KAR-2 does not display anti-calmodulin activity as tested in calmodulin-activated phosphodiesterase assay (Horváth *et al*. 2005). Thus, we suggested that the calmodulin antagonist activity of the bis-indoles might be an important cause of the side effect displayed in complex biological systems, and the lack of calmodulin antagonist activity of KAR-2 could contribute to its low toxic side-effect profile.

The results obtained by studies carried out at the molecular level may provide a plausible explanation for the distinct *in vivo* anti-tumour activities of KAR-2 and vinblastine (Orosz *et al*. 1999). A quantitative measure of this activity is the T/C percentage value of lifespan, the survival factor of the drug-treated animal as compared with the controls. Higher T/C percentage values were obtained with KAR-2 in P388 murine leukaemia or Ehrlich ascites mouse carcinoma, demonstrating the superiority of KAR-2 relative to vinblastine (Orosz *et al*. 1999; Comin-Anduix *et al*. 2001). At single administration of KAR-2, the T/C values were approximately 200 per cent. Therefore, KAR-2 was found to be more effective *in vivo* in terms of anti-tumour efficacy at considerably higher doses relative to vinblastine (Comin-Anduix *et al*. 2001). On the other hand, our comparative studies performed with KAR-2 and its mother molecule, vinblastine, in systems of different complexity (animals, cell lines, cell-free extract and isolated proteins) showed that the toxicity of KAR-2 is much lower than that of vinblastine, although their affinities to the *vinca* site of tubulin are similar (Orosz *et al*. 2006 and references therein). This finding raises the possibility that the primary target of vinblastine may not be the microtubule system, or more probably it does not distinguish between the mitotic and interphase microtubule network.

## 3. Unique bioavailability of KAR-2

The use of bis-indoles in clinical chemotherapy is limited not only owing to their undesirable toxic side effects, but also owing to their reduced bioavailability due to the secretion processes mediated by transporters such as P-glycoprotein (Shalinsky *et al*. 1993; Ferguson *et al*. 2005). The efflux process acts as a barrier for absorption, which is one of the reasons for the poor intestinal permeabilities of these drugs. For the evaluation of the biopharmaceutical and pharmacokinetic characteristics of KAR-2 as compared with vinblastine, and the feasibility of its oral administration, we elaborated an appropriate system and performed comparative studies with Caco-2 cancer cell line and *in situ* animal experiments on the gastrointestinal tract of rat, resembling the human one, and these new data are presented as follows.

The Caco-2 cell culture model (Hidalgo *et al*. 1989) was selected as it has been proven to be a good model of human absorption (Lennernas 1997) and is useful for studying drug secretion (Karlsson *et al*. 1993). Caco-2 cells also express a variety of ATP-binding cassette proteins (or ABC transporters) such as P-glycoprotein (or ABCB1) and MRPs (or ABCC1-6) (Gutmann *et al*. 1999; Eneroth *et al*. 2001; Taipalensuu *et al*. 2001).

Cell monolayers were grown as described previously (Hu *et al*. 1994*a*,*b*). Cell monolayers were prepared by seeding 400 000 cells per insert (MILLICEL-PCF, surface area 4.2 cm^2^, 3 μm pore size). Cell culture was maintained at 37°C under 90 per cent humidity and 5 per cent CO_2_. The *in vitro* study was developed when confluence was reached, 19–22 days post-seeding. The integrity of each cell monolayer was checked by measuring its transepithelial electrical resistance (TEER) value, which was typically 500–750 Ω cm^2^ in our experiments. Cell monolayers with TEER values lower than 420 Ω cm^2^ were not used. Hank's balanced salt solution (9.8 g l^−1^), supplemented with NaHCO_3_ (0.37 g l^−1^), 4-(2-hydroxyethyl) piperazine-1-ethanesulphonic acid (HEPES; 5.96 g l^−1^) and glucose (3.5 g l^−1^) at pH 7 was used to prepare the dosing solution and for the receptor chamber.

The drug solution was loaded into the donor side and buffer was added to the receiver side of each cell monolayer. The six-well plate containing the cell monolayers was put into an orbital environmental shaker, which was maintained at a constant temperature (37°C) and agitation rate (50*g*) for the duration of the transport experiments. Four samples of 200 μl each were taken at 30 min intervals, from the receiver side, and replaced by fresh buffer, for the permeation profile; moreover, two samples of 200 μl each were taken from the donor side, at the beginning and the end of the assay, for the mass balance calculation.

Transport studies were performed in both directions, from apical-to-basolateral (A-to-B) and from basolateral-to-apical (B-to-A) sides. The donor drug concentrations were varied from 5 to 1000 μM and from 25 to 500 μM for vinblastine and KAR-2, respectively.

**Figure 1:**
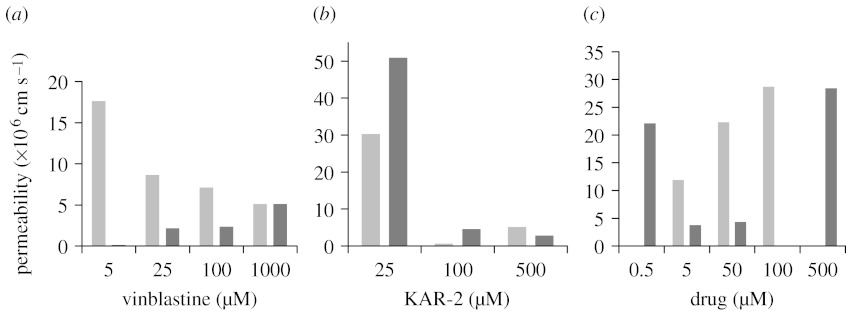
Permeabilities and absorption of KAR-2 and vinblastine in (*a*,*b*) Caco-2 cell line and (*c*) rat model. (*a*,*b*) Caco-2 cell line was provided by Dr Ming Hu (Washington State University, Pullman). Confluent cell monolayers were grown in six-well plates as described elsewhere (Hu *et al*. 1994*a*,*b*). Transport studies were performed in both directions, from apical-to-basolateral (A-to-B, dark grey bars) and from basolateral-to-apical (B-to-A, light grey bars) sides, by loading the drug solution in the donor chamber and taking samples in the acceptor one. The donor drug concentration ranged from 5 to 1000 μM and from 25 to 500 μM for vinblastine and KAR-2, respectively. The apparent unidirectional permeability (*P*_app_) was obtained according to the following equation: (d*Q*/d*t*)/(*SC*), where d*Q*/d*t* is the apparent appearance rate of drug in the receiver side; *S* is the surface area of the monolayer (4.2 cm^2^); and *C* is the concentration in the donor side. (*c*) Absorption rate coefficients (*k*_app_) for each initial concentration were obtained by nonlinear regression of the remaining concentrations in intestinal lumen versus time using an exponential equation. The *k*_app_ values were converted into permeability ones (*P*_app_) via *P*_app_=*k*_app_*R*/2, where *R* is the effective radius of the intestinal segment, calculated as area/volume ratio (Gonzalez-Alvarez *et al*. 2007). Vinblastine, light grey bars; KAR-2, dark grey bars.

The permeabilities (*P*_app_) of the drugs were evaluated from the ratio of the apical-to-basolateral (*P*_AB_) and basolateral-to-apical (*P*_BA_) fluxes at the different drug concentrations. The apparent unidirectional permeability (*P*_app_) was obtained according to the following equation:

(3.1) $$\frac{\text{d}Q/\text{d}t}{SC}=P_{\text{app}},$$

where d*Q*/d*t* is the apparent appearance rate of drug in the receiver side; *S* is the surface area of the monolayer (4.2 cm^2^); and *C* is the concentration in the donor side. The flux term, d*Q*/d*t*, was calculated from the linear regression of amounts in the receiver chamber versus time. As shown in figure 1*a*, when transport experiments were carried out at increasing concentrations, the results showed in the case of vinblastine that *P*_appAB_ increased and *P*_appBA_ decreased on increasing the donor concentration, resulting in a statistically significant efflux ratio (*P*_appBA_/*P*_appAB_>1) at all concentrations except 1000 μM (ratio=1). By contrast, in figure 1*b*, for KAR-2 the efflux ratio (*P*_appBA_/*P*_appAB_) is lower than 1 at relatively low drug concentration, but then increased to 1.8 (500 μM).

The finding that *P*_app_ ratio is high at low drug concentrations, and it decreases on increasing the concentration of vinblastine, indicates the presence of a secretion process. The apparent unidirectional permeability is the result of the sum of the diffusional component (*P*_diff_) and the contribution of the carrier-mediated process (*P*_efflux_). In the apical-to-basolateral direction, the transporter is acting as a barrier to permeation, i.e. *P*_app_=*P*_diff_−*P*_efflux_. At lower drug concentration the contribution of the secretion component is high, decreasing *P*_app_, but at higher drug concentrations, when the transporter is saturated, its contribution becomes negligible and thus *P*_app_ is essentially *P*_diff_. In the basolateral-to-apical experiments, the same phenomenon is observed but in this case the efflux carrier is working in the transport direction, thus *P*_app_=*P*_diff_+*P*_efflux_. Consequently, the apparent permeability is higher at lower drug concentration (when *P*_efflux_ is meaningful) and becomes smaller as the drug concentration increases because *P*_efflux_ becomes negligible. At higher drug concentration, if the transporter is completely saturated, the ratio between *P*_appBA_ and *P*_appAB_ should become 1. The characteristic of the transport process was found to be entirely different for KAR-2, as shown in figure 1*b*. The *P*_app_ ratio was significantly lower than 1 up to 100 μM concentration, which could be attributed to the high permeability and absorption of KAR-2 owing to the fact that KAR-2 is not a substrate for the P-glycoprotein and/or could indicate the presence of a carrier working in the absorptive direction. However, at higher KAR-2 concentrations both influx and efflux decreased 5- to 10-fold, and virtually no active transport of this drug could be detected. The mechanism responsible for this phenomenon is unclear. Nevertheless, the finding that KAR-2 apparently does not interact with P-glycoprotein, in contrast to vinblastine and several other bis-indoles published so far, is an indication of its unique absorption feature originating from its lack of interaction with efflux transporters.

To test this hypothesis, experiments in male Wistar rats were performed. The permeability values of vinblastine in the rat are consistent with a P-glycoprotein substrate and with the results obtained in the cell models.

The absorption experiments were performed using an *in situ* loop technique previously described by Doluisio *et al*. (1969). The procedures used throughout the experiments were previously validated in our laboratory to adapt them to our experimental conditions. The study was approved by the Scientific Committee of the Faculty of Pharmacy and followed the guidelines described in the EC Directive 86/609, the Council of the Europe Convention ETS 123 and Spanish national laws governing the use of animals in research (Real Decreto 223/1988, BOE 67, 18-3-98: 8509-8511). Male Wistar rats weighing 280–320 g were used. After overnight fast with access to water, rats were anaesthetized with thiopental sodium (60 mg kg^−1^ intraperitoneally). A midline abdominal incision was made. The intestinal segment was manipulated carefully in order to minimize any intestinal blood supply disturbances. The bile duct was tied to avoid drug enterohepatic circulation and to limit the presence of bile salts in the lumen. Studies employed the entire small intestine (length of approx. 100 cm). Proximal ligatures of the duodenal and ileal regions were placed approximately 1 cm from the pylorus and 2 cm above the ileocaecal junction. A catheter was tight up at both intestinal ends and connected to a glass syringe by the use of a stopcock-type valve. Under this set-up, the intestinal segment is an isolated compartment and the drug solution can be perfused and tested with the help of the syringes and the stopcock valve.

The drug test solutions (5–100 μM vinblastine and 0.5–500 μM KAR-2) were prepared in an isotonic saline matrix adjusted to pH 7 with 1 per cent (v/v) Sörensen's phosphate buffer. Drug solutions were prewarmed at 37°C in advance. The drug solution was perfused into the loop and then the entire intestine was restored into the abdominal cavity. The body temperature was maintained during anaesthesia by heating with a lamp. Samples were withdrawn every 5 min for 30 min. To correct for variation in drug luminal concentration over time due to water reabsorption, the remaining fluid at 30 min was recovered and volume was measured. Rats were killed humanely at the end of experimentation. In order to separate solid components (e.g. mucus, intestinal contents) from drug solution, samples were centrifuged for 5 min at 5000 r.p.m. (1530*g*), and then quantified by high-performance liquid chromatography.

For each initial concentration, *in situ* rat perfusion data of vinblastine and KAR-2 were analysed in terms of an apparent first-order absorption rate constant. The apparent first-order rate constants were obtained from the expression,

(3.2) $$C=C_{0}{\text{e}}^{-k_{\text{app}}t},$$

where *C* is the drug concentration remaining in the lumen; *k*_app_ is the apparent absorption rate constant; and *C*_0_ is the initial drug concentration. Data applied to equation (3.2) were previously corrected for water reabsorption, *k*_app_ was estimated using nonlinear regression with WinNonlin v. 5.2 and it was transformed into a permeability value (*P*_app_) via *P*_app_=*k*_app_*R*/2, where *R* is the effective radius of the intestinal segment, calculated as area/volume ratio (Gonzalez-Alvarez *et al*. 2007).

The results obtained in these *in situ* absorption studies are presented in figure 1*c*. In the case of vinblastine, the permeability significantly increased on increasing the donor concentration. The KAR-2 permeability characteristic is more complex. It decreased when KAR-2 concentration was increased from 0.5 to 50 μM, but at 500 μM concentration, rather surprisingly, the permeability value increased again. While the permeability profile clearly showed characteristics that are representative of secretion of vinblastine, KAR-2 displayed a complex absorption pattern, which may suggest the simultaneous presence of transporters for secretion and absorption. The most important result is that, even at the highest concentration, KAR-2 permeability is very close to that of vinblastine, thus ensuring at least the same absorbability as vinblastine but a much better penetration into cancer cells or a broader tissue distribution because its affinity for P-glycoprotein is almost negligible. Although we do not know the mechanism responsible for the unique bioavailability properties of KAR-2, nevertheless, our data suggest that KAR-2 displays a much better absorption profile and penetration characteristics than its mother drug, vinblastine, which could ensure appropriate oral absorption during clinical treatment.

## 4. KAR-2 counteracts with TPPP/p25 in cancer cells

To evaluate the mechanism associated with the distinct toxic side effects of KAR-2 and vinblastine, we have performed comparative studies based upon the visualization of the interphase and mitotic microtubule network of two human cancerous cell lines, cervical carcinoma (HeLa) and neuroblastoma (SH-SY-5Y) after drug treatment. The smallest effective concentration of KAR-2, 0.5 μM, was chosen from the concentration range of 0.1–4 μM. Both bis-indoles were added to the complete medium of the growing cells for 24 hours in order to let all the cells go through one mitotic phase of the cell cycle. For the fluorescent microscopic analysis, cells were grown on 12 mm coverslips; otherwise cells were grown as described previously (Lehotzky *et al*. 2004). For monitoring the architecture of the microtubules, immunocytochemistry was carried out. Drug-treated and non-treated control cells were fixed in ice-cold methanol for 10 min and immunostained with monoclonal anti-tubulin antibody (DM1A, Sigma), followed by FITC (fluorescein isothiocyanate) conjugated anti-mouse IgG secondary antibody (Jackson Laboratories) to visualize the microtubule network. Nuclei were stained by DAPI (4′,6-diamidino-2-phenylindole). Images were prepared with a Leica DMLS epifluorescent microscope using an Apofluor objective with 100× magnification.

**Figure 2:**
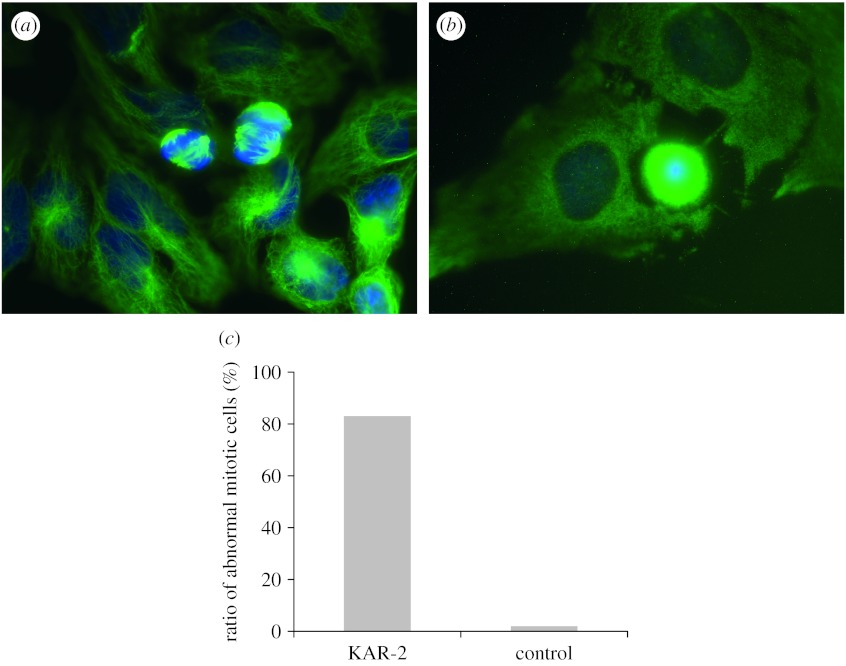
Effect of KAR-2 and vinblastine on microtubular ultrastructures. HeLa cells were maintained and treated as described previously (Lehotzky *et al*. 2004). The drugs were added ((*a*) 0.5 μM KAR-2 and (*b*) 0.5 μM vinblastine) to the medium and incubated for 24 hours. Control cells were handled in a similar manner except that no drug was added to the medium. The samples were immunostained with anti-tubulin antibody (monoclonal DM1A, detected as green) to visualize the microtubular network. Blue indicates nuclei stained by DAPI. (*c*) The ratio of mitotic cells bearing aberrant spindles versus all mitotic cells in the KAR-2-treated and the non-treated sample.

In the vinblastine-treated cells, neither the interphase nor the spindle microtubule network could be seen; the tubulin showed a homogeneous distribution in the cytosol due to the complete disassembly of both microtubule networks (figure 2*b*). On the contrary, in the KAR-2-treated sample the interphase microtubules appeared to be normal, unaffected by the drug (figure 2*a*); while the spindle microtubules were aberrant in most cases. In the vinblastine-treated samples, the cells lost their ability to form spindle microtubules, and no array of spindle fibres could be detected, while in the case of KAR-2 treatment spindles originated from more than two poles, leading to an asymmetrical segregation of the chromosomes due to the abnormal mitotic process. In comparison, the microtubule spindles of the control cells were nucleated at the opposite poles of the spindles to form a bipolar astral array, which were positioned and orientated to provide a proper axis to the equal cell division (data not shown). This ultrastructural arrangement is necessary for the division of the nucleus, a process in which the chromosomes are propelled by attachment to the mitotic spindle, a bundle of microtubules; otherwise the cell division is arrested.

**Scheme 1:**
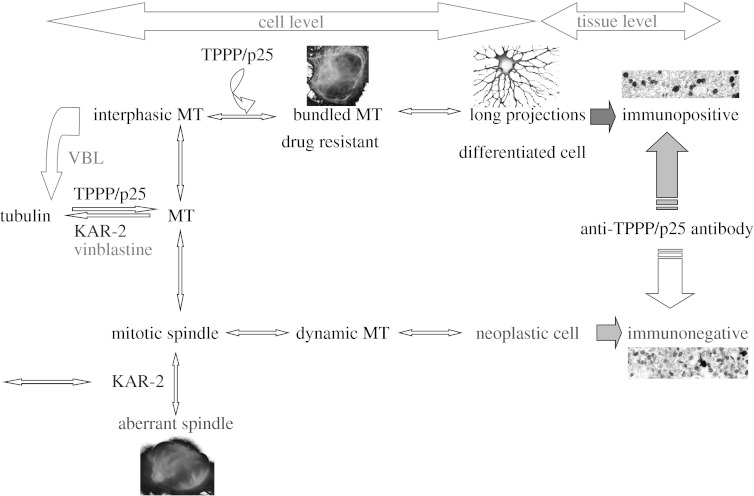
Tentative scheme for the effects of KAR-2 and TPPP/p25 on microtubule (MT) systems. Both vinblastine and KAR-2 inhibit the taxol-promoted tubulin polymerization and cause microtubule disassembly *in vitro*. At cell level, KAR-2 induces the formation of aberrant mitotic spindles without affecting the interphase microtubule network. On the contrary, vinblastine preferentially damages the interphase microtubule system. TPPP/p25 stabilizes the microtubule network, resulting in vinblastine-resistant bundled microtubules and the formation of long projections in differentiated oligodendrocytes. At tissue level, normal brain cells show TPPP/p25 immunopositivity while neoplastic cells are immunonegative.

To quantify the effect of the two drugs on the mitotic spindle formation, we counted the abnormal and normal mitotic cells on random microscopic fields in the KAR-2-treated and the non-treated control samples. These data showed that 0.5 μM KAR-2 drastically arrested the cell cycle process at the mitotic phase (figure 2*c*), because the percentages of multipolar spindles were found to be 83 and 2 per cent for the KAR-2-treated and the non-treated mitotic cells, respectively. Therefore, our data showed that, although vinblastine and KAR-2 showed comparable anti-microtubular activity *in vitro* in the tubulin polymerization assay (Orosz *et al*. 1997*a*), KAR-2 displayed a specific *in vivo* anti-microtubule effect: it selectively targeted the mitotic spindles (scheme 1).

The experiments with neuroblastoma cells (SH-SY-5Y) led to results that had high impact in the light of data obtained with HeLa cells. The neuroblastoma cells were more sensitive to drug treatment than the HeLa cells. Thus, the concentrations of the drugs used were decreased for the treatments. In fact, the concentrations were chosen to correspond to their IC_50_ values of viability (IC_50_ of viability for KAR-2 and vinblastine are 316 and 5 nM, respectively; Orosz *et al*. 1999). Consequently, treatment with 5 nM vinblastine did not cause complete disassembly of the microtubule network and approximately 30 per cent of the mitotic cells were normal, while the microtubule network of the cells in the interphase were damaged severely (Comin-Anduix *et al*. 2001) and only less than 10 per cent of the KAR-2-treated cells in mitosis were normal.

The data of the two sets of experiments show that the severe anti-microtubular activity of vinblastine preferentially targets the interphase microtubules, while KAR-2 has no effect on the microtubule network in the interphase even at 0.5 μM concentration. At the same time, KAR-2 attacks selectively the mitotic spindles, resulting in aberrant multipolar spindle formation, a unique characteristic expected from a specific anti-mitotic drug.

**Figure 3:**
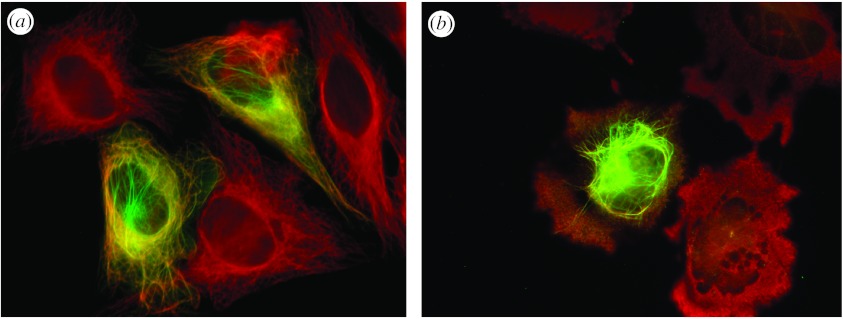
Effect of vinblastine on TPPP/p25-stabilized microtubular network. HeLa cells were transiently transfected with *EGFP-TPPP* plasmid, expressing the green fluorescent protein coupled with TPPP/p25 (Lehotzky *et al*. 2004). After 22 hours post-transfection, (*b*) vinblastine or (*a*) vehicle was added to the medium and incubated for a further 2 hours. Samples were immunostained with anti-tubulin antibody (monoclonal DM1A, detected as red) to visualize tubulin/microtubules by fluorescent microscopy. The microtubular network is preserved in an EGFP-TPPP/p25-expressing cell shown in (*b*) in spite of the vinblastine treatment.

The severe anti-microtubule activity of vinblastine is extensively offset by TPPP/p25. The HeLa cell line, which does not contain endogenous TPPP/p25, was transiently transfected with *EGFP-TPPP* construct, in order to express fusion green fluorescent TPPP/p25 (Lehotzky *et al*. 2004). For visualization of the microtubule network, immunostaining with anti-tubulin monoclonal antibody (DM1A, Sigma) was performed. Previously we demonstrated that TPPP/p25 was perfectly aligned with the microtubule network at low expression level (figure 3*a*). To investigate the effect of TPPP/p25 on the stability of the microtubule network, vinblastine-induced microtubule disassembly was tested.

The vinblastine concentration in the complete medium of the transiently transfected HeLa cells was 50 nM in the last 2 hours of the 24 hour transfection period. Control cells were not transfected but treated with vinblastine. In these cells, the microtubule network collapsed completely; a uniform distribution of depolymerized microtubules was visible in the cytosol (figure 3*b*, red). However, in the EGFP-TPPP/p25-expressing cells the microtubule network appeared to be resistant to the drug effect (figure 3*b*), as indicated by the TPPP/p25-bundled microtubule network, which was located around the perinuclear region. These data show that the presence of TPPP/p25-counteracts the effect of vinblastine due to its very active bundling activity, resulting in stabilization of microtubules.

The microtubule network should be reorganized during the cell cycle process specifically at the anaphase when the microtubule network is still disassembled. Accordingly, the amount of soluble cytosolic TPPP/p25 was found to be high in this phase, and the microtubules also showed a diffuse picture; while throughout the interphase perfect colocalization of the TPPP/p25 and the microtubule was detected (Lehotzky *et al*. 2004). The question we addressed was whether the TPPP/p25 impeded the cell cycle process via its microtubule stabilization effect. Could it deplete the mitosis of the cancer cell?

**Figure 4:**
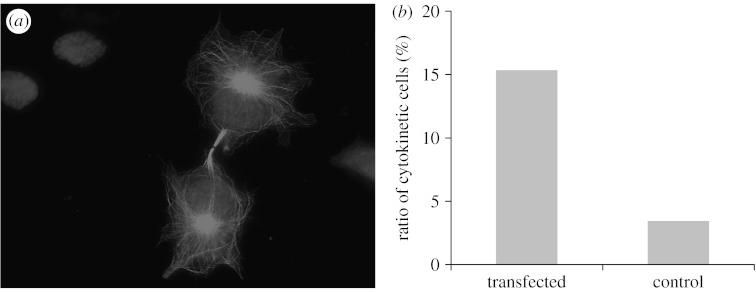
Effect of TPPP/p25 on the cell cycle. HeLa cells were transiently transfected with EGFP-TPPP plasmid, expressing the green fluorescent protein coupled with TPPP/p25 (Lehotzky *et al*. 2004). (*a*) An example of an EGFP-TPPP/p25-expressing cell in cytokinesis (nuclei stained with DAPI). (*b*) The ratio of cells in cytokinesis as compared with the total counted cell number. Transfected and control cells were visualized and counted by fluorescent and phase contrast microscopy, respectively. For comparison, 200 transfected and 400 control cells were investigated for the presence of the cleavage furrow.

Thus, we investigated the effect of EGFP-TPPP/p25 expression on the cell cycle of cancerous cells. EGFP-TPPP/p25 expressed in HeLa cells was also aligned along the microtubule network in the interphase of HeLa cells, and enriched at the region of the centrosomes (data not shown). A modest accumulation of the green fluorescent protein (GFP) over the mitotic spindle is visible in metaphase as well as in anaphase. The amount of EGFP-TPPP/p25 associated with the microtubules increased during cytokinesis and this accumulation was specifically extensive over the microtubule bundles of the cleavage furrow (figure 4*a*). This bundling activity of TPPP/p25, which has also been demonstrated in living cells (Lehotzky *et al*. 2004), can result in the stabilization of the furrow structure. Quantitative comparison of the transfected and non-transfected (control) cells showed that, while the ratio of cleavage furrow-bearing cells (cells in cytokinesis versus total number of cells) was 3–5 per cent in the control sample counted on random microscopic fields by phase contrast microscopy, this ratio increased to 15 per cent in the case of the transfected cells (figure 4*b*). A plausible explanation for this finding is that TPPP/p25 stabilizes the microtubule-based furrow ultrastructure, which impedes the separation of the two daughter cells, by which the rate of proliferation may decrease in cancerous cells. Therefore, stimulation of TPPP/p25 expression or deregulation of TPPP/p25 expression at the protein level could display severe anti-proliferative activity. This hypothesis is compatible with our previous observations showing that TPPP/p25 injection into cleavage *Drosophila* embryos inhibits mitotic spindle assembly and nuclear envelope breakdown without affecting other cellular events (Tirián *et al*. 2003). Thus, understanding the role of TPPP/p25 in cell division might open a new perspective in cancer research.

## 5. Availability of TPPP/p25 as a potential biomarker in oncological diagnosis

TPPP/p25 was independently identified as a brain-specific protein occurring mainly in oligodendrocytes and in the neuropil (Takahashi *et al*. 1993). Later on, TPPP/p25 was found to be present in the oligodendroglial cells of the white matter and perineuronal oligodendroglial cells in the cortex of the normal human brain, in addition to faint immunostaining of the neuropil (Kovács *et al*. 2007). The premyelinating cells are formed from oligodendrocyte progenitor cells during brain development and differentiate into myelin-forming oligodendrocytes (Skjoerringe *et al*. 2006). This differentiation process is accompanied by the appearance of long arborized projections as well as the expression of myelin basic protein (MBP; Akiyama *et al*. 2002). We hypothesized that TPPP/p25, which impedes cell division and stabilizes microtubule network, could play a role in the differentiation of the oligodendrocyte progenitor cells.

Recently, we have screened a number of human and other eukaryotic cells, including cancerous cells, searching for the presence of endogenous TPPP/p25, without success. Because this protein is primarily expressed in oligodendrocytes, we searched for its expression in CG-4 cells, a permanent cell line derived from primary cultures of bipotential oligodendrocyte type 2 astrocyte (O-2A) progenitor cells of the rat central nervous system (CNS) glial precursors, which could be differentiated into oligodendrocytes (Louis *et al*. 1992). To our knowledge, this is the first study performed on cells endogeneously expressing TPPP/p25 at protein level.

**Figure 5:**
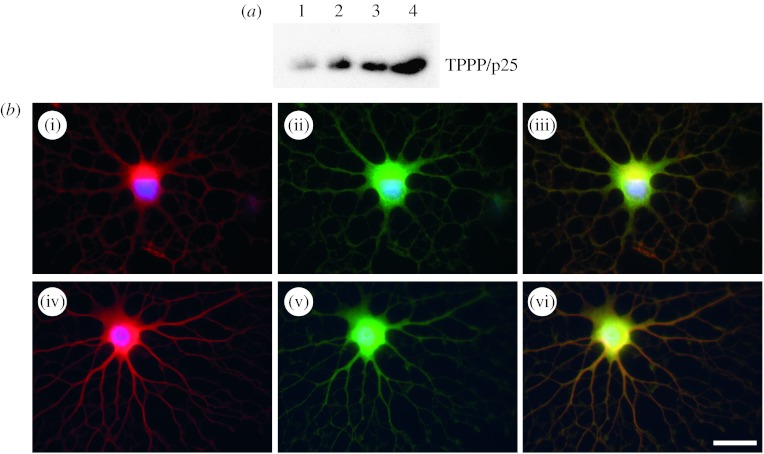
(*a*) Expression of TPPP/p25 in CG-4 cells and (*b*) distribution of TPPP/p25 and marker proteins in differentiating oligodendrocytes. (*a*) CG-4 progenitor cells were plated on poly-l-ornithine-coated vessels in 70/30 proliferation medium (1), and in oligo medium (2, 3, 4) to promote *in vitro* differentiation of progenitors to prematured oligodendrocytes. Samples were prepared before (1) and at second (2), third (3) and fourth (4) days after induction of differentiation. For the relative determination of the TPPP/p25 expression, western blot using a rat polyclonal antibody raised against the full length of TPPP/p25 was done (Kovács *et al*. 2007). The amount of the protein loaded into the SDS–PAGE gel was 2 μg in each case. (*b*) CG-4 cells were plated on poly-l-ornithine-coated glass coverslips in oligo medium and were differentiated for 4 days. The formaldehyde-fixed cells were visualized by immunofluorescence using a rat polyclonal antibody raised against the full length of TPPP/p25. Mouse monoclonal anti-MBP (Covance) and mouse monoclonal anti-class IV β-tubulin (Sigma) antibodies were used as additional primary antibodies. Primary antibodies were detected by anti-rat IgG Cy2-conjugated antibody depleted against mouse serum (for TPPP/p25, Jackson Laboratories) and anti-mouse IgG Texas Red-conjugated antibody depleted against rat serum (for MBP and class IV β-tubulin, Jackson Laboratories) to ensure no cross-reactivity of the conjugated secondary antibodies: MBP (red; i, iii), class IV β-tubulin (red; iv, vi) and TPPP (green; ii, iii, v, vi). Scale bar, 10 μM.

In one set of experiments, we investigated the TPPP/p25 amount by western blot before and after induction of differentiation of precursor cells. Samples were withdrawn during the experimental time course in RIPA buffer supplemented with protease inhibitor mix (Sigma products). Equal protein amounts were loaded onto sodium dodecyl sulphate polyacrylamide gel electrophoresis (SDS–PAGE), and, following the separation of the protein bands, they were transferred onto polyvinylidene fluoride (PVDF) membrane (Millipore, 0.45 μm pore size) by standard procedures. The membrane was blocked by 5 per cent fat-free milk powder dissolved in phosphate-buffered saline. The specific antibody to detect TPPP/p25 was raised against human recombinant protein as described in detail elsewhere (Kovács *et al*. 2007). Then the blot was incubated with HRPO (horseradish peroxidase) conjugated anti-rat IgG (Sigma). Chemiluminescent substrate (Immobilon Western, Millipore) was used to detect the TPPP/p25-specific signal on the blot. The western blot data showed that the level of TPPP/p25 expression was relatively low in dividing progenitor cells, which gradually increased during the course of the differentiation (figure 5*a*).

In the second set of experiments, we investigated the TPPP/p25 expression during the maturation of oligodendrocytes by fluorescent microscopy. To follow the differentiation process, in addition to the morphological changes of the cells, the expression of the MBP and class IV β-tubulin was detected by immunofluorescent microscopy as well as by using specific antibodies.

Progenitor culture was trypsinized, then 25 000 cells were plated onto a poly-l-ornithine-coated coverslip; next the cells were propagated in 70/30 or oligo medium (Louis *et al*. 1992). In oligodendrocyte progenitors, a faint TPPP/p25 and MBP immunopositivity were visualized by immunofluorescent microscopy after formaldehyde fixation (70/30 medium, data not shown). However, the immunopositivity increased dramatically for both proteins in differentiating cells (oligo medium, figure 5*b*(i)–(iii)). This finding unambiguously indicated that the expression of TPPP/p25 and the MBP increased concomitantly during the differentiation of the oligodendrocytes, in addition to the morphological changes of the prematured cells. Moreover, we found that the two proteins showed similar subcellular distribution at the periphery of the cells, especially on the long arborized projections of prematured oligodendrocytes. This observation was supported by the fact that in the case of cold methanol fixation the signal of endogenous TPPP/p25 weakened strongly in samples of differentiated CG-4 cells (data not shown). These findings combined with that obtained in brain tissue by immunohistochemistry (Skjoerringe *et al*. 2006) may suggest that TPPP/p25 is a membrane-associated protein. We also observed co-immunostaining of TPPP/p25 and class IV β-tubulin in differentiating oligodendrocytes, providing evidence for the colocalization of these two proteins on the long projections of the cells at the cellular periphery of the oligodendrocytes (figure 5*b*(iv)–(vi)).

Our present data obtained with the CG-4 cells reveal that TPPP/p25 is virtually not present in progenitor cells and strongly upregulated in differentiating oligodendrocytes. Recently, a microarray analysis of gene expression of oligodendrocyte progenitor cells expressing the A2B5^+^ marker and differentiating O4^+^ oligodendrocytes revealed that TPPP/p25 transcript level increased 20- to 30-fold during the progression from A2B5 to the O4 stage (Nielsen *et al*. 2006). Real-time polymerase chain reaction was also employed to confirm the upregulation of TPPP/p25 transcript. At the protein level, TPPP/p25 is expressed specifically in the perinuclear cytoplasm of myelinating oligodendrocytes and as a constituent of myelin (Skjoerringe *et al*. 2006) in rat brain. Together, these findings show that the differentiated oligodendrocytes express TPPP/p25 in significant amount. Because undifferentiated mitotic precursors do not normally express TPPP/p25, we examined whether TPPP/p25 was expressed in neoplastic cells found in different brain tumours, including oligodendrogliomas.

**Figure 6:**
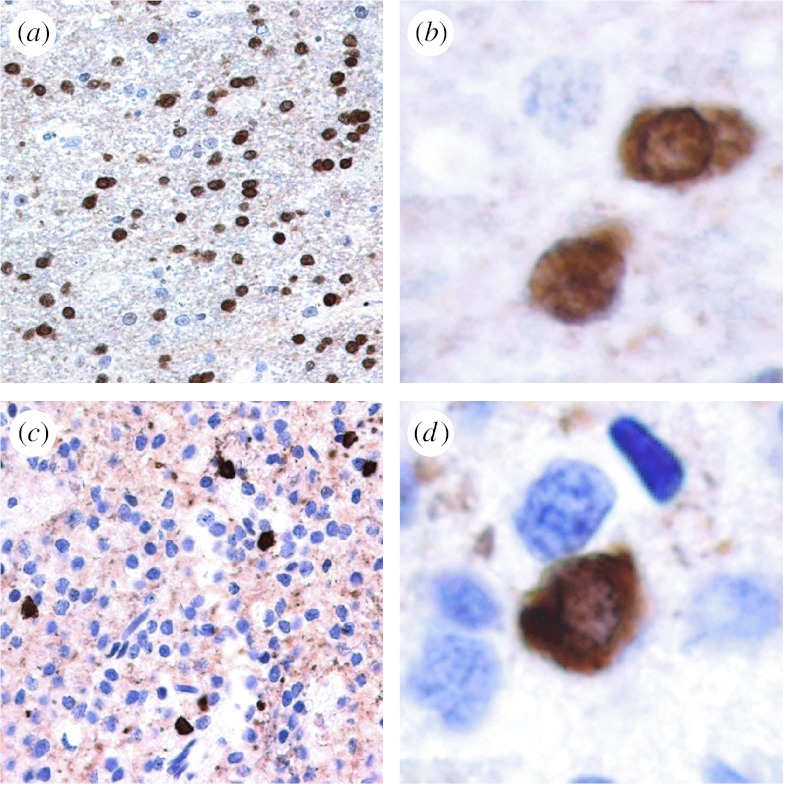
Immunostaining for TPPP/p25 in white matter of the temporal lobe. For immunohistochemistry, tissue specimens were fixed in phosphate-buffered 5.5 per cent solution of formaldehyde and embedded in paraffin. Slides underwent epitope retrieval in 0.01 M citrate buffer (pH 6.0) for 10 min. We used a rat polyclonal antibody (1 : 200) raised against the full length of TPPP/p25 (Kovács *et al*. 2007). Detection of immunostaining was performed using the Envision kit (DakoCytomation, Glostrup, Denmark), and diaminobenzidine was used as chromogene. (*a*,*b*) Non-neoplastic normal tissue and (*c*,*d*) oligodendroglial tumour tissue (original magnifications: (*a*,*c*) ×200 and (*b*,*d*) ×600).

Our cohort of samples comprised several histological types of brain tumours. Immunohistochemical screening confirmed immunoreactivity in oligodendrocytes of non-neoplastic CNS tissue (figure 6*a*,*b*). None of the brain tumours showed unequivocal immunopositivity for TPPP/p25. However, in diffusely infiltrating oligodendroglial neoplasms, we found individual cells with immunoreactivity most likely representing pre-existing oligodendrocytes (figure 6*c*,*d*). We concluded that TPPP/p25 is a marker of differentiated, non-neoplastic cells (Preusser *et al*. 2007). On the one hand, this finding, together with previous observations (Skjoerringe *et al*. 2006), supports the notion that oligodendrogliomas arise from immature, undifferentiated glial precursors rather than from neoplastic transformation of mature oligodendrocytes. On the other hand, one cannot exclude the possibility that, during the process of neoplastic transformation, oligodendrocytes lose their ability to express TPPP/p25, or this feature is suppressed by other, as yet unidentified, genetic or epigenetic factors. This would be compatible with the anti-mitotic activity of TPPP/p25 shown in *Drosophila* embryos (Tirián *et al*. 2003).

In this *in vivo* experiment, isolated TPPP/p25 was injected into cleavage *Drosophila* embryos expressing tubulin–GFP fusion protein. Microinjection of proteins into the posterior pole of cleavage embryos has been found to be a powerful method to study the functions of proteins that affect cell cycle progression because the synchronized nuclear divisions 9–14 commence at the surface of the shared egg cytoplasm, allowing visualization of the effects of the injected protein on several nuclei and spindles. These experimental data revealed that TPPP/p25 inhibited mitotic spindle assembly and nuclear envelope breakdown without affecting other cellular events such as centrosome replication and separation, microtubule nucleation by the centrosomes and nuclear growth. TPPP/p25 was found to be only slightly expressed during the embryonic period in rat brain; its expression increased extensively in the first weeks after birth, then gradually increased until 1–2 years of age (Takahashi *et al*. 1993). The observation referring to the expression pattern of TPPP/p25 along with our present finding obtained with oligodendrocyte cells indicates that TPPP/p25 probably plays a physiological role during cell differentiation.

The role of TPPP/p25 in oncogenesis and tumour progression needs further clarification. Most likely this is a complex scenario, which may be cell specific and may be different at the level of gene and protein expression. Recent studies identified gain of 5p15.33, including *TPPP/p25* gene, to be associated with the progression of bladder and non-small cell lung cancer (Yamamoto *et al*. 2007; Kanga *et al*. 2008). In the first case, the authors also indicated that this gain is associated with not only high histological grade but also advanced pathological stage in bladder cancer. Supposedly, the amplification of *TPPP/p25* gene may confer a growth advantage to urothelial cells. In the latter case, the gain of genes was observed in early stages of the cancer. However, it is not proved in either case that the gene copy changes correlate with protein or even mRNA expression. On the contrary, it was shown that both the gene copy number and the expression of several miRNAs are significantly increased in various types of cancer, including ovarian cancer, breast cancer and melanoma (Zhang *et al*. 2006). In the case of 26 miRNAs, the increases were shared among all the three cancer types investigated. These miRNAs involved miR-1, which was predicted to regulate TTTP/p25 expression. This finding has several implications. First, it indicates that TPPP/p25 may also be expressed in non-CNS tissue as recently reported in mouse (Acevedo *et al*. 2007). Second, it suggests that cells may behave differently during neoplastic transformation and that gene and protein expression may influence this process distinctly. TPPP/p25 may have anti-mitotic activity as suggested by our present and previous studies (Tirián *et al*. 2003), and its over-expression can cause aberrant tubulin assemblies (Hlavanda *et al*. 2002), which, conversely, lead to pathological division in neoplastic cells. These processes merit further studies in oligodendroglial tumours.

Both KAR-2 and TPPP/p25 appear as anti-mitotic agents; however, the mechanism by which they express their effects are different. KAR-2 directly targets mitotic spindles, resulting in arrest of cell division at the M-phase of the cell cycle. Because a relatively small fraction of the cells stay in this state with respect to the interphase one, the undesired toxic effect of KAR-2 is relatively low with respect to the other bis-indoles used in chemotherapy. TPPP/p25 is apparently absent or present only at low concentration in proliferating cells, such as cancer cells; when its expression at protein level is upregulated, the differentiation of the cells is initiated, probably because the cell enters the quiescent G_0_ state from G_1_ and they remain quiescent for long periods of time, as is the case for neurons.

## References

[bib1] Acevedo K., Li R., Soo P., Suryadinata R., Sarcevic B., Valova V.A., Graham M.E., Robinson P.J., Bernard O. (2007). The phosphorylation of p25/TPPP by LIM kinase 1 inhibits its ability to assemble microtubules. Exp. Cell Res.

[bib2] Akiyama K., Ichinose S., Omori A., Sakurai Y., Asou H. (2002). Study of expression of myelin basic proteins (MBPs) in developing rat brain using a novel antibody reacting with four major isoforms of MBP. J. Neurosci. Res.

[bib3] Altmann K.-H., Gertsch J. (2007). Anticancer drugs from nature—natural products as a unique source of new microtubule-stabilizing agents. Nat. Prod. Rep.

[bib4] Bhat K.M., Setaluri V. (2007). Microtubule-associated proteins as targets in cancer chemotherapy. Clin. Cancer Res.

[bib5] Comin-Anduix B., Agell N., Bachs O., Ovádi J., Cascante M. (2001). A new bis-indole, KARs, induces selective M arrest with specific spindle aberration in neuroblastoma cell line SH-SY5Y. Mol. Pharmacol.

[bib6] Doluisio J.T., Billups N.F., Dittert L.W., Sugita E.T., Swintosky J.V. (1969). Drug absorption I: An *in situ* rat gut technique yielding realistic absorption rates. J. Pharm. Sci.

[bib7] Dutcher S.K. (2001). The tubulin fraternity: alpha to eta. Curr. Opin. Cell Biol.

[bib8] Eneroth A., Astrom E., Hoogstraate J., Schrenk D., Conrad S., Kauffmann H.M., Gjellan K. (2001). Evaluation of a vincristine resistant Caco-2 cell line for use in a calcein AM extrusion screening assay for P-glycoprotein interaction. Eur. J. Pharm. Sci.

[bib9] Ferguson R.E. (2005). Intrinsic chemotherapy resistance to the tubulin-binding antimitotic agents in renal cell carcinoma. Int. J. Cancer.

[bib10] Gonzalez-Alvarez I., Fernandez-Teruel C., Casabo-Alos V.G., Garrigues T.M., Polli J.E., Ruiz-Garcia A., Bermejo M. (2007). *In situ* kinetic modelling of intestinal efflux in rats: functional characterization of segmental differences and correlation with *in vitro* results. Biopharm. Drug Dispos.

[bib11] Gutmann H., Fricker G., Torok M., Michael S., Beglinger C., Drewe J. (1999). Evidence for different ABC-transporters in Caco-2 cells modulating drug uptake. Pharm. Res.

[bib12] Hidalgo I.J., Raub T.J., Borchardt R.T. (1989). Characterization of the human colon carcinoma cell line (Caco-2) as a model system for intestinal epithelial permeability. Gastroenterology.

[bib13] Hlavanda E., Kovács J., Oláh J., Orosz F., Medzihradszky K.F., Ovádi J. (2002). Brain-specific p25 protein binds to tubulin and microtubules and induces aberrant microtubule assemblies at substoichiometric concentrations. Biochemistry.

[bib14] Horváth I., Harmat V., Perczel A., Pálfi V., Nyitray L., Nagy A., Hlavanda E., Náray-Szabó G., Ovádi J. (2005). The structure of the complex of calmodulin with KAR-2: a novel mode of binding explains the unique pharmacology of the drug. J. Biol. Chem.

[bib15] Hu M., Chen J., Tran D., Zhu Y., Leonardo G. (1994a). The Caco-2 cell monolayers as an intestinal metabolism model: metabolism of dipeptide Phe-Pro. J. Drug Target.

[bib16] Hu M., Chen J., Zhu Y., Dantzig A.H., Stratford R.E., Kuhfeld M.T. (1994b). Mechanism and kinetics of transcellular transport of a new β-lactam antibiotic loracarbef across an intestinal epithelial membrane model system (Caco-2). Pharm. Res.

[bib18] Kanga J.U., Kooa S.H., Kwona K.C., Parka J.W., Kimb J.M. (2008). Gain at chromosomal region 5p15.33, containing TERT, is the most frequent genetic event in early stages of non-small cell lung cancer. Cancer Genet. Cytogenet.

[bib17] Karlsson J., Kuo S.M., Ziemniak J., Artursson P. (1993). Transport of celiprolol across human intestinal epithelial (Caco-2) cells: mediation of secretion by multiple transporters including P-glycoprotein. Br. J. Pharmacol.

[bib20] Kovács G.G. (2004). Natively unfolded tubulin polymerization promoting protein TPPP/p25 is a common marker of α-synucleinopathies. Neurobiol. Dis.

[bib19] Kovács G.G., Gelpi E., Lehotzky A., Hoftberger R., Erdei A., Budka H., Ovádi J. (2007). The brain-specific protein TPPP/p25 in pathological protein deposits of neurodegenerative diseases. Acta Neuropathol.

[bib21] Lehotzky A., Tirián L., Tőkési N., Lénárt P., Szabó B., Kovács J., Ovádi J. (2004). Dynamic targeting of microtubules by TPPP/p25 affects cell survival. J. Cell Sci.

[bib22] Lennernas H. (1997). Human jejunal effective permeability and its correlation with preclinical drug absorption models. J. Pharm. Pharmacol.

[bib23] Louis J.C., Magal E., Muir D., Manthorpe M., Varon S. (1992). CG-4, a new bipotential glial cell line from rat brain, is capable of differentiating *in vitro* into either mature oligodendrocytes or type-2 astrocytes. J. Neurosci. Res.

[bib24] Luduena R.F. (1998). Multiple forms of tubulin: different gene products and covalent modifications. Int. Rev. Cytol.

[bib25] MacRae T.H. (1992). Towards an understanding of microtubule function and cell organization: an overview. Biochem. Cell Biol.

[bib26] Makarov A.A., Tsvetkov P.O., Villard C., Esquieu D., Pourroy B., Fahy J., Braguer D., Peyrot V., Lafitte D. (2007). Vinflunine, a novel microtubule inhibitor, suppresses calmodulin interaction with the microtubule-associated protein STOP. Biochemistry.

[bib27] Nielsen J.A., Maric D., Lau P., Barker J.L., Hudson L.D. (2006). Identification of a novel oligodendrocyte cell adhesion protein using gene expression profiling. J. Neurosci.

[bib28] Orosz F., Kovács J., Löw P., Vértessy B.G., Urbányi Z., Ács T., Keve T., Ovádi J. (1997a). Interaction of a new bis-indol derivative, KAR-2 with tubulin and its antimitotic activity. Br. J. Pharmacol.

[bib29] Orosz F., Vértessy G.B., Salerno C., Crifo C., Capuozzo E., Ovádi J. (1997b). The interaction of a new anti-tumor drug, KAR-2 with calmodulin. Br. J. Pharmacol.

[bib30] Orosz F. (1999). New semisynthetic vinca alkaloids: chemical, biochemical and cellular studies. Br. J. Cancer.

[bib32] Orosz F., Kovács G.G., Lehotzky A., Oláh J., Vincze O., Ovádi J. (2004). TPPP/p25: from unfolded protein to misfolding disease: prediction and experiments. Biol. Cell.

[bib31] Orosz F., Horváth I., Ovádi J. (2006). New anti-mitotic drugs with distinct anti-calmodulin activity. Mini Rev. Med. Chem.

[bib33] Preusser M., Lehotzky A., Budka H., Ovádi J., Kovács G.G. (2007). TPPP/p25 in brain tumours: expression in non-neoplastic oligodendrocytes but not in oligodendroglioma cells. Acta Neuropathol.

[bib34] Shalinsky D.R., Jekunen A.P., Alcaraz J.E., Christen R.D., Kim S., Khatibi S., Howell S.B. (1993). Regulation of initial vinblastine influx by P-glycoprotein. Br. J. Cancer.

[bib35] Skjoerringe T., Lundvig D.M.S., Jensen P.H., Moos T. (2006). P25α/tubulin polymerization promoting protein expression by myelinating oligodendrocytes of the developing rat brain. J. Neurochem.

[bib36] Sudakin V., Yen T.J. (2007). Targeting mitosis for anti-cancer therapy. BioDrugs.

[bib37] Taipalensuu J. (2001). Correlation of gene expression of ten drug efflux proteins of the ATP-binding cassette transporter family in normal human jejunum and in human intestinal epithelial Caco-2 cell monolayers. J. Pharmacol. Exp. Ther.

[bib38] Takahashi M., Tomizawa K., Fujita S.C., Sato K., Uchida T., Imahori K. (1993). A brain-specific protein p25 is localized and associated with oligodendrocytes, neuropil, and fiber-like structures of the CA3 hippocampal region in the rat brain. J. Neurochem.

[bib39] Tirián L., Hlavanda E., Oláh J., Horváth I., Orosz F., Szabó B., Kovács J., Szabad J., Ovádi J. (2003). TPPP/p25 promotes tubulin assemblies and blocks mitotic spindle formation. Proc. Natl Acad. Sci. USA.

[bib40] Vincze O. (2006). Tubulin polymerization promoting proteins (TPPPs): members of a new family with distinct structures and functions. Biochemistry.

[bib41] Yamamoto Y. (2007). Gain of 5p15.33 is associated with progression of bladder cancer. Oncology.

[bib42] Zhang L. (2006). MicroRNAs exhibit high frequency genomic alterations in human cancer. Proc. Natl Acad. Sci. USA.

